# Beyond the Lab: Scaling Wearables for Real‐World Impact through Partnerships and Implementation

**DOI:** 10.1002/alz70863_110891

**Published:** 2025-12-23

**Authors:** Christa Studzinski

**Affiliations:** ^1^ Ontario Brain Institute, Toronto, ON Canada

## Abstract

Despite the advancement of wearable technologies, widespread adoption and implementation of these potentially transformative technologies to support aging individuals, including those with mild cognitive impairment (MCI), Alzheimer's disease, and related dementias (ADRD), has yet to be achieved. This barrier to adoption has prevented us from fully realizing the potential transformative impact wearables could have – both in the delivery of compassionate and accessible dementia care and in improving outcomes for aging individuals. This session will share lessons learned from the Ontario Brain Institute's journey to co‐design and launch a program called CORTEX (Community‐led Real‐world neuroTech Experience) that empowers community groups and people with lived experience in testing, implementing, and scaling wearable technology.

We will share how the initial concept for CORTEX emerged, how the program was initially co‐designed with community groups and people with lived experience, and the learnings from the first pilots conducted in partnership with patient groups. Finally, we will show how the learnings informed the launch of our flagship CORTEX initiative, the Canadian Dementia Registry, which was co‐created in partnership with the Alzheimer Society of Ontario. This registry allows us to (1) understand the earliest stage of the dementia journey diagnosis (including how conducting cognitive assessments in the community, on behalf of primary care practitioners fills an early detection gap), (2) test neurotechnologies in diverse populations and compare them to clinically‐relevant scales (e.g., MoCA), and (3) look at how these technologies can fit into the care pathway in local communities so that we can increase access to the right clinicians and care they need and deserve.

